# The development of a self-management evaluation scale for elderly adults with hypertension based on the capability, opportunity, and motivation-behaviour (COM-B) model

**DOI:** 10.1186/s12877-023-03879-1

**Published:** 2023-04-22

**Authors:** Lirong Wu, Minhui Liu, Chongmei Huang, Jinzhi Yin, Hui Zhou, Hongjuan Hu

**Affiliations:** 1 School of Nursing, Hengyang Medical School, University of South China, Hengyang, 421001 Hunan China; 2 The Affiliated Changsha Central Hospital, Hengyang Medical School, University of South China, Hunan 410013 Changsha, China; 3 Nursing School of Central South University, Changsha, 410013 China; 4 The First Affiliated Hospital, Department of Public Service/Nursing department, Hengyang Medical School, University of South China, Hunan 421001 Hengyang, China

**Keywords:** Elderly hypertension, Self-management, COM-B model, Scale, Reliability, Validity

## Abstract

**Background:**

Using accurate assessment tools to assess patients in clinical practice is important to mining influencing factors and implementing interventions. However, most evaluation tools for the self-management of elderly patients with hypertension lack a theoretical basis and wide applicability, which makes the intervention effect insignificant.

**Methods:**

Based on the Capability, Opportunity, and Motivation-Behaviour (COM-B) model, combined with literature review and qualitative research, a questionnaire item pool was initially formulated; then the initial items were screened and adjusted through expert consultation and pre-testing to form an initial scale. A field survey of 450 elderly hypertensive patients was then performed using the initial scale to test the reliability and validity of the scale. Cronbach’s alpha, test–retest reliability and composite reliability were used to test the reliability of the scale, and the validity of the scale was evaluated from two aspects: content validity and construct validity. The evaluation results of the content validity of the scale by experts were used as the content validity index; the results of exploratory factor analysis and confirmatory factor analysis were used as the structural validity index to further verify the model structure of the scale and develop a formal scale.

**Results:**

The final self-management scale included 4 dimensions and 33 items. The Scale-Content Validity Index was 0.920. Exploratory factor analysis extracted four factors that explained 71.3% of the total variance. Cronbach’s alpha of the formal scale was 0.867, test–retest reliability was 0.894, and composite reliability of the 4 dimensions were within 0.943 ~ 0.973. Confirmatory factor analysis showed the scale had good construct validity.

**Conclusions:**

The Self-management Capability, Support and Motivation-Behaviour scale for elderly hypertensive patients has good reliability and validity, providing a tool for medical staff to evaluate the self-management level of elderly hypertensive patients.

## Background

Hypertension is strongly associated with cardiovascular diseases, and has become the leading risk factor for premature death [1]. In 2019, the worldwide prevalence of hypertension in the age group 30–79 was more than 33.0%, translating into a worldwide population of more than 12.8 million people with hypertension [2]. China has a high incidence of primary hypertension, with hypertension prevalence rates of 13.6%, 18.8%, 25.2%, and 27.9% found in nationwide epidemiological investigations in 1991, 2002, 2012, and 2017, respectively [3–6]. This prevalence rate has been steadily increasing. The elderly population in China is particularly affected, with those aged ≥ 60 years old accounting for 53.2% of elderly patients with hypertension, or up to 2.45 million individuals [7, 8].

Hypertension is an independent risk factor for stroke and cardiovascular diseases [9, 10]. The annual direct economic burden is as high as 20.2 billion Yuan [11], which imposes a tremendous burden on the family and society. Studies show that hypertension can increase the risk of mobility, cognitive, and emotional disorders and other diseases in elderly patients, thus affecting their ability to perform daily activities, and reducing their independence and quality of life [12, 13]. It is particularly urgent to effectively prevent and control hypertension. In China, a policy titled “Medium and Long-Term Plan for the Prevention and Treatment of Chronic Diseases (2017–2025)” [14] proposed that patients with cardiovascular and cerebrovascular diseases, hypertension, and other chronic diseases should be included in screening intervention and health management projects to improve residents’ life expectancy, and effectively control the burden of chronic diseases.

Considering the long-term and incurable nature of hypertension, the “Guidelines for the Education of Chinese Hypertensive Patients” and the theme of the 2013 World Health Day emphasized [15]: the treatment of hypertension cannot only rely on medical treatment, but it should also depend on the prevention and control of hypertension. It is necessary to strengthen the prevention and control education of hypertension, and adhere to the management of their blood pressure for a long time. However, the current self-management level of elderly hypertensive patients in China is not ideal enough [16, 17]. The prevalence, awareness, treatment, and control rates of hypertension are 61.1%, 40.2%, 33.9%, and 11.3% respectively, which are far lower than those of Western developed countries [8]. A study has shown that effective self-management can reduce blood pressure, improve the quality of life of patients and promote the effective use of medical resources [18]. The self-management of hypertension is important for the prevention and treatment of this condition in China.

Accurate evaluation tools are a prerequisite in clinical practice and a key to discovering influencing factors and implement interventions [19]. After a review of the extant literature, we found that most of the evaluation tools for self-management in elderly patients with hypertension were designed by the researchers according to the research purpose, lacked theoretical basis and did not have broad applicability (Table 1) [20–23], making it difficult for medical staff to understand the patient’s condition, and the intervention measures taken might not be effective.

**Table 1:** Features of hypertension self-management tools

Scale	Reliability and validity	Advantages	Disadvantages
Chronic Disease Self-Management Study Measures	Cronbach’s α of each dimension: 0.72–0.91, test–retest reliability: 0.65–0.80	Recognized, with good reliability and validity, applicable to all patients with chronic diseases	Not specific. The self-management assessment only involves exercise, cognitive symptom management, and communication with doctors; and the content of the assessment is not comprehensive
Hypertension Patients of Self-Management Behavior Rating Scale	Cronbach’s α of each dimension: 0.757–0.911, Cronbach’s α of total scale: 0.914, Content Validity Index (CVI): 0.82–0.94, Overall scale CVI: 0.91	Currently widely used in hypertension self-management	Assessment content is not comprehensive and the test–retest reliability of the scale has not been examined
Hypertension Self-Management Scale	Cronbach’s alpha for total scale: 0.854, total halving: 0.856, Overall scale CVI: 0.976	It involves the content of the risk factor dimension, management dimension is low	There is no theoretical basis for the development of the scale, and the reliability of the risk factor
Hypertension Self-Profile	Cronbach’s alpha for the total scale: 0.950, test–retest reliability: 0.918, scale CVI: 0.83–1.00	The scale has a clear theoretical. framework, and its content addresses motivational factors	The expression of items in the scale is too specialized, the evaluation of a total of 60 items is time-consuming, and it is mainly used to evaluate the self-care aspects of hypertensive patients

Up to date, the Knowledge-Attitude-Practice (KAP) model and the Information-Motivation-Behavioral (IMB) model have been widely used in the self-management of hypertension in China [24, 25]; however, they only focus on the aspects of individual knowledge and individual motivation, ignoring the external environment and personal motivation. The role of the individual’s own ability on behavior change has not been fully explored and utilized to maximize the intervention effect. The Capability, Opportunity, and Motivation-Behaviour (COM-B) model is the only behavior change model [26] to link the influencing factors of behavior and intervention strategies, providing clear guidance for behavior analysis and intervention design. It emphasizes analyzing the factors and mechanisms influencing individual behavior, focusing on the capability (psychological and physical abilities, such as knowledge and skills), opportunity (physical and social environment, such as medical and family support), and motivation (reflexivity and spontaneity, such as self-efficacy) which affect individual behaviors, making up for the inadequacy of the existing theoretical analysis. At home and abroad, it is mainly used for the promotion of individual health behaviors and the analysis of behavioral disorders and motivational factors [27–29]. It can be a comprehensive method to explore the relevant factors of individual behavior to provide a suitable theoretical framework. Therefore, this study uses the COM-B model as the theoretical framework to develop tools that can comprehensively and systematically evaluate the self-management of elderly hypertensive patients, and provide a reference for formulating more effective nursing interventions.

## Methods

### Theoretical framework

**Fig. 1:**
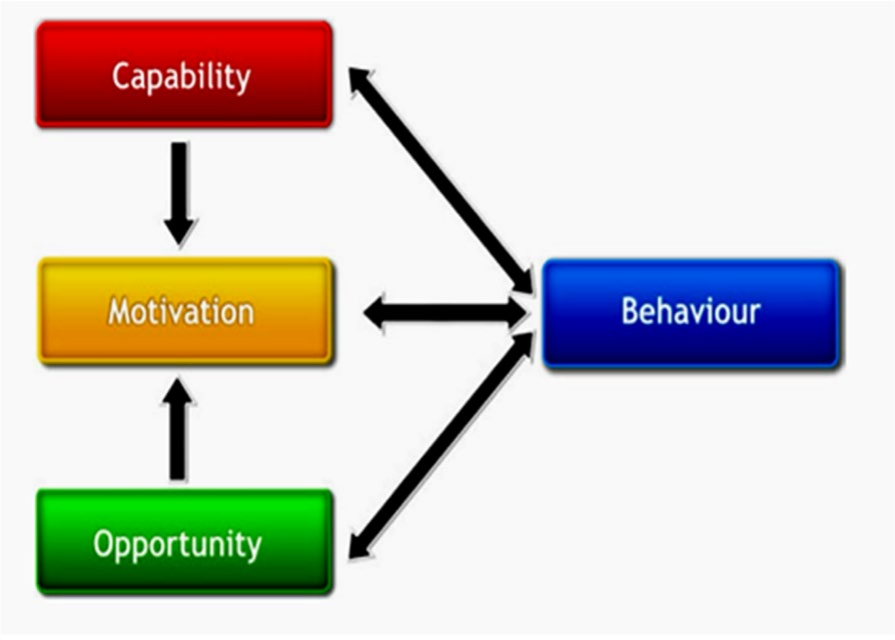
COM-B Model

**Fig. 2:**
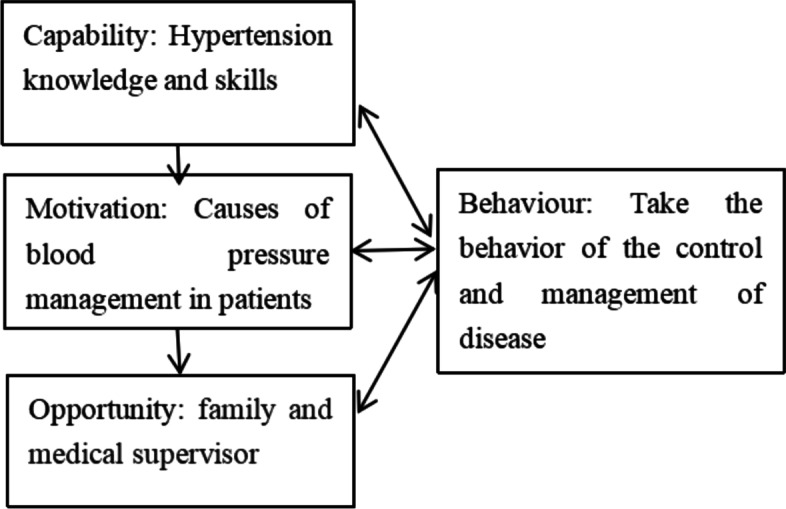
COM-B model in this study freame diagram

This study utilizes the COM-B model, a theoretical framework for behavior change that illustrates how capability, opportunity, and motivation interact to produce behavior change (Fig. 1). The model’s three main aspects provide researchers with a comprehensive and clear direction to analyze behavior mechanisms in specific situations and the first step in designing behavior interventions. The study's theoretical framework was based on existing research and a literature review (Fig. 2).

### Participants and setting

This was a cross-sectional study, which used convenience sampling to recruit elderly hypertensive patients between August 2021 and December 2021. A total of 450 participants were recruited from two community health service centers in Hunan, China. Inclusion criteria were as follows: aged 60 years or elderly; diagnosed with primary hypertension; been receiving antihypertensive drugs therapy for more than six months; living in the community for more than six months; informed and agreed to participate in this study. Exclusion criteria were as follows: people with severe mental (such as schizophrenia, anxiety, manic depression, depression, etc.) and communication disorders; serious physical acute and chronic diseases. According to the principle of Kendall sample size calculation, when testing the reliability and validity of the scale, the sample should not be less than 5–10 times the number of entries [30]. This study was approved by the Ethics Committee of Nanhua University and ethical approval was obtained from participating communities before data collection. Participation was voluntary and could be withdrawn before data analysis. Return of the completed questionnaire was taken as a consent to participate.

### Procedures

This study involved two stages: 1) Development of the scale and 2) Testing its reliability and validity.

#### Step one: Development of the scale

**Fig. 3:**
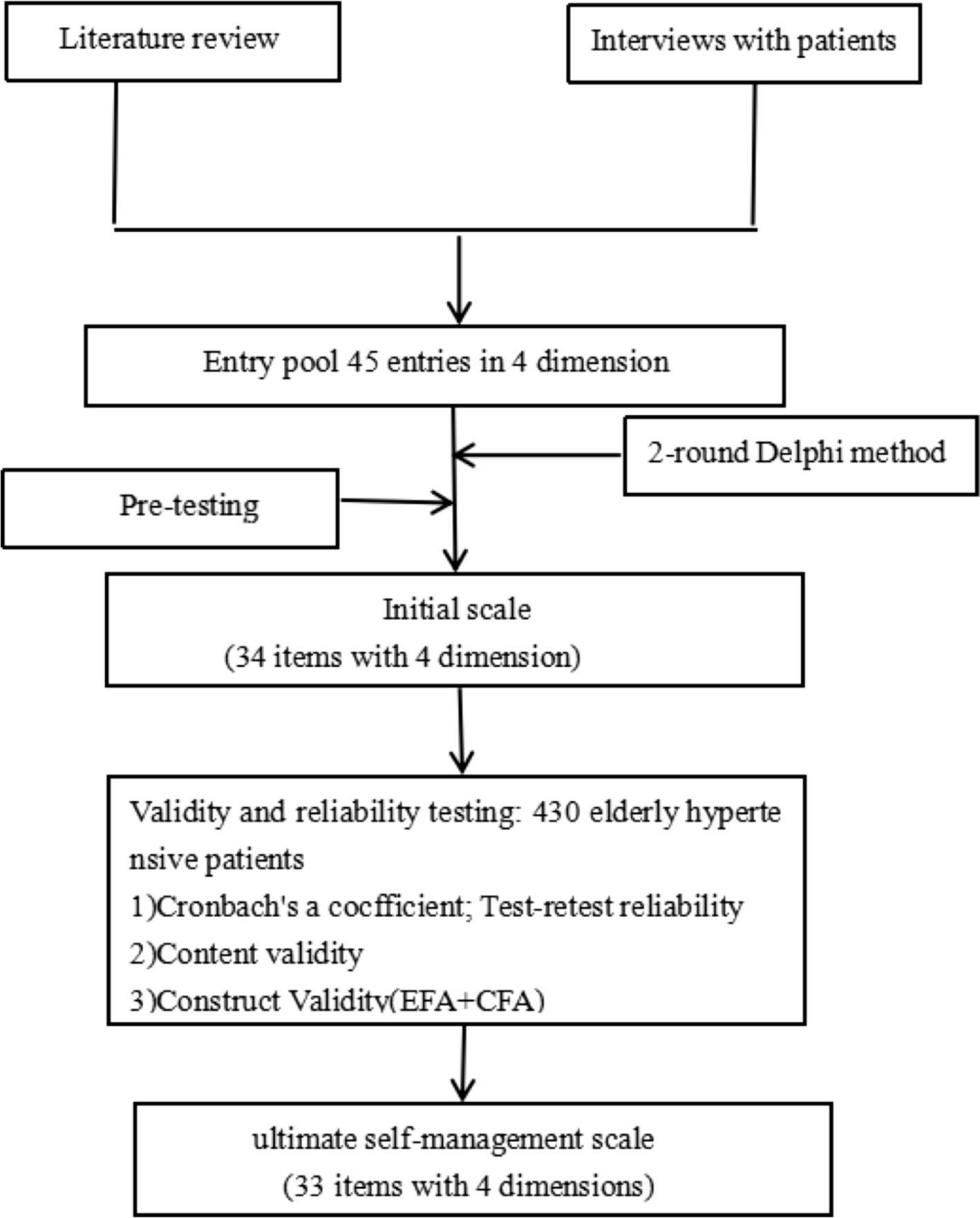
Flow diagram of the development process of the scale

Guided by COM-B theory, first, a literature review, the interview method, and the Delphi method were used to preliminarily formulate the questionnaire of self-management capability, opportunity, motivation-behaviour of elderly hypertensive patients scale. Second, the questionnaire expression through the pre-testing was used to form the initial scale. After item selection with several statistical analyses, an ultimate self-management scale with 4 domains and 33 items was completed (Fig. 3).

First, we searched CNKI, Wanfang, PubMed, Cochrane Library, Web of Science and other databases to review the current domestic and foreign literature on the content and application status of the COM-B model framework [31, 32], and refer to the “Chinese guidelines for the Management of Hypertension in the Elderly 2019” and the “2018 Chinese guidelines for the Management of hypertension” [33, 7] to provide theoretical support and practical guidance for the scale, and then initially formed a pool of 35 entries in 4 dimensions based on literature review and group discussions.

Second, guided by the COM-B model, convenience sampling was used to select 13 elderly hypertensive patients who were treated by a community health service center in Hengyang for interviews. A total of three themes and six sub-themes were extracted. Theme one, “Deficient in self-management capability” includes two sub-themes: vague understanding of hypertensive diseases and insufficient ability of self-monitoring blood pressure. Theme two, “Insufficient self-management motivation” includes two sub-themes: lack of motivational factors for blood pressure management and pressure due to complications. Theme three, “Lack of self-management support” includes two sub-themes: insufficient family support and the need for medical support to be strengthened. Combining the aforementioned methods, 4 dimensions and 45 items were formed after the group discussions.

Third, the research team selected 16 experts from six provinces including Jiangsu, Beijing, Guangzhou, Hunan, Sichuan, and Hebei. Experts were primarily engaged in chronic disease management, cardiovascular medicine, psychology, and other fields. On the basis of the literature review and group discussion, the researcher designed the expert consultation form, mainly including letters to experts, expert consultation forms, naming questions of this scale, and expert information forms. The expert consultation ends when the expert opinions were basically consistent. The reliability of the expert consultation was tested by using four indicators: the positive coefficient of experts; the coefficient of expert authority; Kendell’s W, and the mean and coefficient of variation of the project importance assignment; the judgment result of the item, and add, delete, or modify the item.

Fourth, using purposive sampling method, we selected 20 elderly hypertensive patients from a community service center, in Hengyang City, for pre-testing, testing the readability of the questionnaire, and collecting opinions on the questionnaire expression.

#### Step two: Testing the reliability and validity

The validity and reliability of the final scale was assessed using responses from 430 elderly hypertensive patients.

### Instrument

① General Information Questionnaire: It was designed by the researchers on the basis of the literature review, and it included age, gender, educational level, marital status, occupation type, monthly family income CNY (¥), medical insurance, and duration of hypertension.

② “Self-management COM-B of elderly hypertensive patients” initial scale with 34 items: All items in this scale adopted positive scoring. Likert’s 5-level scoring method was used for capability dimension (1 = strongly disagree, 5 = strongly agree); and also for support, motivation, and behaviour dimensions (1 = very inconsistent, 5 = very consistent). Among them, the higher the scores of capability, support, and behaviour dimensions, the higher the self-management level of elderly hypertensive patients. The motivation dimension was taken to be a categorical variable which mainly reflects if the self-management behavior of patients is affected by spontaneous motivation or reflexive motivation.

### Statistical analysis

All the data were encoded and input into the computer. For statistical analysis, SPSS 22.0 and AMOS 21.0 software were used. Sample and expert general information were analyzed using numbers, means, and percentages.

Content validity: The calculation was mainly based on expert evaluation results. Content validity is divided into Item-Content Validity Index (I-CVI) and Scale-Content Validity Index (S-CVI). I-CVI calculation method: number of experts who rated 3 or 4 points by total number of experts; S-CVI calculation method: number of items which were awarded 3 or 4 points by all experts by total number of scale items. I-CVI > 0.78, S-CVI > 0.80, indicating that the scale has good content validity [30].

Reliability: ① Cronbach’s α coefficient was calculated for each factor and the overall scale to test the consistency within the scale. ② Test–retest reliability: 20 patients were selected for re-testing, their scores were recorded, and the correlation coefficients of the two measurements were calculated to assess the test–retest reliability. The statistically acceptable reliability coefficient should be > 0.70 [34]. ③ Composite reliability: generally, AVE > 0.5 and CR > 0.750 are the standard values [35].

Exploratory Factor Analysis (EFA) Bartlett sphericity test and Kaiser–Meyer–Olkin (KMO) test should be performed before EFA measurement to determine whether the questionnaire items are suitable for the factor analysis. Bartlett’s test of Sphericity with *p* < 0.05 and a KMO value of 0.60 were suitable for running the EFA. The number of factors was selected according to the Kaiser criterion and the Cattell’s scree test [36]: ① Kaiser criterion recommends selecting entries with factor load > 0.40, and at least 3 or more items of factors. ② Cattell’s scree test recommends that all factors above the elbow or break in the plot should be retained.

Confirmatory Factor Analysis (CFA): CFA was conducted to further verify the structure validity model of the questionnaire. To conduct parameter estimation, we used the maximum likelihood method and Pearson correlation, given that the data in this study followed a normal distribution. Model fit indices, such as χ^2^/df, CFI (Comparative Fit Index), GFI (Goodness-of-Fit Index), IFI (Incremental Fit Index), TLI (Tucker–Lewis Index), RMSEA (Root Mean Square Error of Approximation), PGFI (Parsimony-goodness-of-fix index), PNFI (Parsimony-adjusted, NFI) were used for model goodness-of-fit assessment. The goodness of fit indices in structural equation modeling are typically evaluated by the proximity of GFI, IFI, CFI, and TFI to 1. Values above 0.900 indicate a very ideal model, while values above 0.800 indicate a good model. An RMSEA less than 0.100 represents acceptable model fit, and PGFI and PNFI values greater than 0.50 are indicative of a reasonable fit, as noted in [37].

## Results

### Development of the scale

#### Expert characteristics of the sample

Table 2 shows expert characteristics. The average age was 43.80 years (SD = 7.4), and the average length of service was 20.73 years (SD = 10.13); senior professional titles accounted for 75.0% of the experts.

**Table 2:** Expert characteristics (*N* = 16)

Variables	Category	Number	(%)
Gender	Male	4	40
	Female	12	60
Age	30–40	5	31
	41–50	9	56
	> 50	2	13
Working years	11–20	8	50
	21–30	6	38
	> 30	2	12
Education	Master	7	44
	PhD	9	56
Job title	Intermediate	4	25
	Advanced	12	75
Career field	Cardiovascular medicine	7	44
	Psychology	2	12
	Chronic disease management	7	44

### Expert consultation result

The return rates of the 2 rounds of expert consultation questionnaires were 100% and 94.0%, respectively. The authority coefficients of experts were 0.87 in round 1 and 0.90 in round 2; and the Kendall’s coordination of coefficients was 0.312 in round 1 and 0.523 in round 2 (*P* < 0.05 for both). The item importance score of the first round of expert consultation was 2.94 ~ 4.88 points, and the coefficient of variation score was 0.07 ~ 0.48 points, the item importance score of the second round of expert consultation was 2.40 ~ 4.87 points, and the coefficient of variation score was 0.07 ~ 0.41 points.

For 45 items of the original scale, after the first round of expert consultation, 13 items were deleted, three items were added, one item was split, and 26 items were modified. The specific modification is as following: ① 10 items were deleted, including “When you have headaches, dizziness and other symptoms, you should see a doctor in time” whose mean value and coefficient of variation of importance and feasibility did not meet the standard; ② Deleted three phrases with insufficient execution and repetitious meaning, such as “The bad attitude of the medical staff made it difficult for me to understand my blood pressure”; ③ Split the item “I know how to control my blood pressure through diet and exercise” into two items; ④ Because of the scale for self-measuring scale, so “your family will prompt you to quit alcohol” in 20 items such as “you” is amended as “I”; ⑤ In addition, an entry about drug skills was added to the capability dimension, an entry about community chronic disease management was added to the support dimension, and an entry about reflexive motivation was added to the motivation dimension, adding 3 items in total; ⑥ Six items such as “ I know how to use the sphygmomanometer to measure blood pressure” were revised linguistically according to expert opinions, and finally four dimensions comprising 36 items were retained.

According to the second round of expert opinion and relevant statistical results, two items were deleted, five items were modified, and four dimensions comprising 34 entries were finally retained. In addition, 14 experts suggested the use of the “Self-Management Capability, Support, Motivation-Behaviour Scale for Elderly Hypertension.” Furthermore, the research object of this study is elderly hypertensive patients, and in the COM-B model, “opportunity” means “support.” Therefore, this study uses “Self-management Capability, Support, Motivation-Behaviour Scale for Elderly Hypertension” as the name of the scale which was developed.

### Pre-testing

A total of 20 elderly hypertensive patients in the community were investigated, and 20 questionnaires were recovered, with an effective recovery rate of 100%. Among them, nine were male and eleven were female (Age 73.20 ± 7.22 years old). The questionnaire was completed within 10 min, and the opinions were mainly centered around the usage of professional terms in the questionnaire, which were difficult to understand. Therefore, after discussion with this research group, the corresponding revisions were made (Table 3) to generating an initial scale of 34 items in four dimensions.

**Table 3:** Pre-testing modification items

Original entry	Modified entry
I know what the main complications of high blood pressure are	I know what hazards high blood pressure can cause
I know what the risk factors for high blood pressure are	I know what causes high blood pressure
I will proactively take steps to relieve anxiety to control blood pressure	I will think about the positives when I get into negative situations or have unhappy thoughts

### Testing the reliability and validity

#### Characteristics of the sample

Among the 450 participants recruited in this study, 430 participants completed the questionnaire. The effective recovery rate was 95.6%. The age ranged from 60 to 91 years, and the mean age was 70.81 ± 7.85 years; there were 230 males and 200 females; 256 people with primary school and junior high school education; 148 people with a disease duration of more than 1–5 years; and 381 people mainly engaged in non-medical work-related occupations before retirement (Table 4).

**Table 4:** Patient characteristics (*N* = 430)

Project	Category	N (%)
Age	60–69	190 (44.2)
	70–79	157 (36.5)
	> = 80	83 (19.3)
Gender	Male	230 (53.5)
	Female	200 (46.5)
Education	Primary and below	122 (28.4)
	Junior high school	134 (31.2)
	High school	111 (25.8)
	Junior college	23 (5.3)
	College degree and above	11 (5.1)
Marital status	Married	334 (77.7)
	Widowed	91 (21.2)
	Unmarried/divorce/other	5 (1.2)
Occupation type	Medical work-related careers	49 (11.4)
	Non-medical work-related careers	381 (88.6)
Monthly family income CNY (¥)	< 2000	46 (10.7)
	2001–4000	139 (32.2)
	4001–6000	158 (36.7)
	> 6000	87 (20.2)
Medical insurance	Resident medical insurance	62 (14.4)
	Employee medical insurance	287 (66.7)
	New Rural Cooperative Medical Scheme	60 (14.0)
	Self-funded	17 (4.0)
	Commercial insurance	4 (0.9)
Duration of hypertension	< 1 years	32 (7.4)
	1–5 years	148 (34.4)
	6–10 years	107 (24.9)
	> 10 years	143 (33.3)

#### Reliability

The Cronbach’s α coefficient for this study was 0.867, which changed slightly after removing an item from one of the four dimensions (Capability, Support, Motivation-Behavior). The test–retest reliability was 0.894, and the group reliability of the 4 dimensions in the metric table ranged from 0.943 to 0.973, all exceeding 0.70 and meeting the acceptable range of combination reliability [35] (Table 5).

**Table 5:** The internal consistency, test–retest reliability and composite reliability of the formal scale

Dimension	Cronbach’s ɑ	Test–retest reliability	AVE	CR
Capability	0.797	0.833	0.645	0.947
Support	0.861	0.776	0.702	0.943
Motivation	0.701	0.777	0.847	0.971
Behaviour	0.866	0.853	0.790	0.973
Total	0.867	0.878	––	––-

#### Content validity

This study invited eight experts to participate in the evaluation of content validity, including three cardiovascular medical diseases, two psychology, and three chronic disease management experts. The average age of the experts was 45.75 ± 9.16 years, the S-CVI was 0.92, and the I-CVIs ranged from 0.88 ~ 1.00.

#### Construct validity

**Fig. 4:**
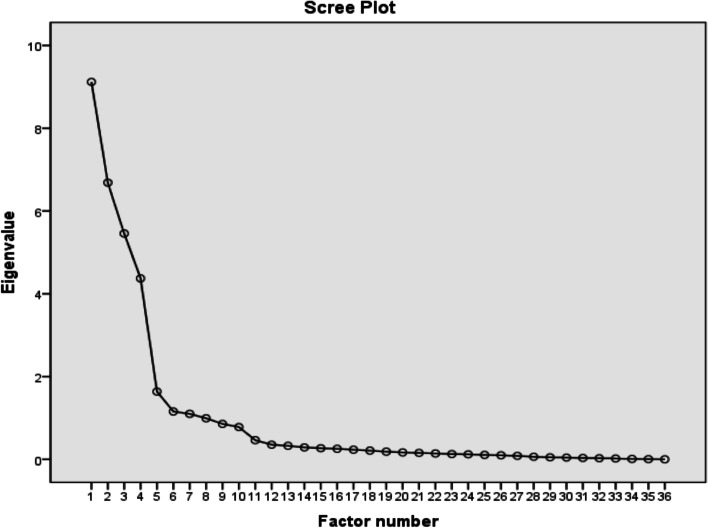
Scree Plot

The value of KMO was 0.823 and the result of Bartlett’s sphericity test was χ^2^ = 11,508.961 (*P* < 0.001), indicating that the sample was suitable for a factor analysis. Using the principal component maximum variance rotation factor analysis method, seven factors were extracted with Eigenvalues > 1 without limiting the number of factors, for which cumulative variance contribution rate was 82.0%. Considering that the COM-B model used in this study mainly includes four dimensions, it was reasonable to limit to four common factors when extracting factors. Therefore, four common factors were limited to carry out the second exploratory factor analysis. The factor cumulative variance contribution rate was 71.2%, of which C1 had a double load phenomenon and was eliminated. The remaining items were then subjected to the third exploratory factor analysis. An inspection of Cattell’s scree plot (Fig. 4) revealed a clear break after the fourth component. The factor loading for each item was above 0.4 without cross-loadings. Therefore, the scale finally retained 4 factors and 33 items (Table 6), which were named as F1: Capability dimension (C1-C10), F2: Support dimension (S1-S7), F3: Motivation dimension (M1-M6), and F4: Behaviour dimensions (B1-B10).

**Table 6:** Results from exploratory factor analysis of the final scale (*N* = 215)

Item	F1	F2	F3	F4
**Capability dimension**				
C3. I know what hazards high blood pressure can cause	.827			
C4. I know what causes high blood pressure	.826			
C9. I know how to control my blood pressure through exercise	.813			
C1.I can tell if my blood pressure is normal	.795			
C11.I know that I should take the medicine according to the instructions prescribed by the doctor	.792			
C8. I know how to control my blood pressure through diet	.777			
C6. I know that I should measure my blood pressure every day	.775			
C5.I know that I should go to the relevant hypertension clinic according to the doctor's advice	.745			
C7. I know how to measure my blood pressure	.744			
C2. I know what the main symptoms of hypertension are	.724			
**Support dimension**				
S3. My family will urge me to quit smoking or limit alcohol		.907		
S1. My family will help me adjust my bad eating habits such as high salt, greasy and heavy flavors		.873		
S4. My family reminds or helps me measure my blood pressure every day		.855		
S6. The chronic disease contract mechanism established by the community (providing one-to-one door-to-door service) is convenient for me to better manage my blood pressure		.853		
S5. My family paid more attention to my blood pressure situation and gave me a lot of confidence to manage my blood pressure		.845		
S2. My family will urge me to keep exercising		.788		
S7. Medical staff will provide me with corresponding blood pressure management measures (medications, exercise, diet, etc.) according to my situation		.732		
**Motivation dimension**				
M1. I started to manage my blood pressure because I learned that poor blood pressure control could cause serious physical and mental problems			.980	
M2. I started to manage blood pressure management based on my own health care			.935	
M4. I started managing my blood pressure because my family and friends told me the importance of blood pressure management			.921	
M3. I started to manage blood pressure through publicizing the knowledge of hypertension and the importance of blood pressure management through medical staff			.900	
M5. I started managing my blood pressure because high blood pressure increased the care burden of my family			.899	
M6. I started to manage my blood pressure because I learned that some patients with hypertension suffered from stroke, cardiovascular diseases and other diseases because they did not manage their blood pressure			.882	
**Behaviour dimension**				
B4. I will start reducing smoking				.947
B5. I will monitor my weight regularly and control my weight myself				.934
B3. I will start to cut down on alcohol				.907
B6. I will take part in physical exercise regularly (for example, 3–5 times a week, 30 min of exercise time each time)				.907
B8. I will actively obtain knowledge about hypertension (such as consulting with medical staff, communicating with other hypertension patients, etc.)				.896
B2. I will eat less salty foods (such as pickles, pickles, kimchi, etc.)				.871
B9. I will take the hypertension medication according to the doctor's instructions				.839
B7. I will think about the positives when I get into negative situations or have unhappy thoughts				.806
B1. I will monitor my own blood pressure daily				.793
B10. I will adjust the time, amount and content of my work (housework) according to my blood pressure				.639

#### CFA

**Fig. 5:**
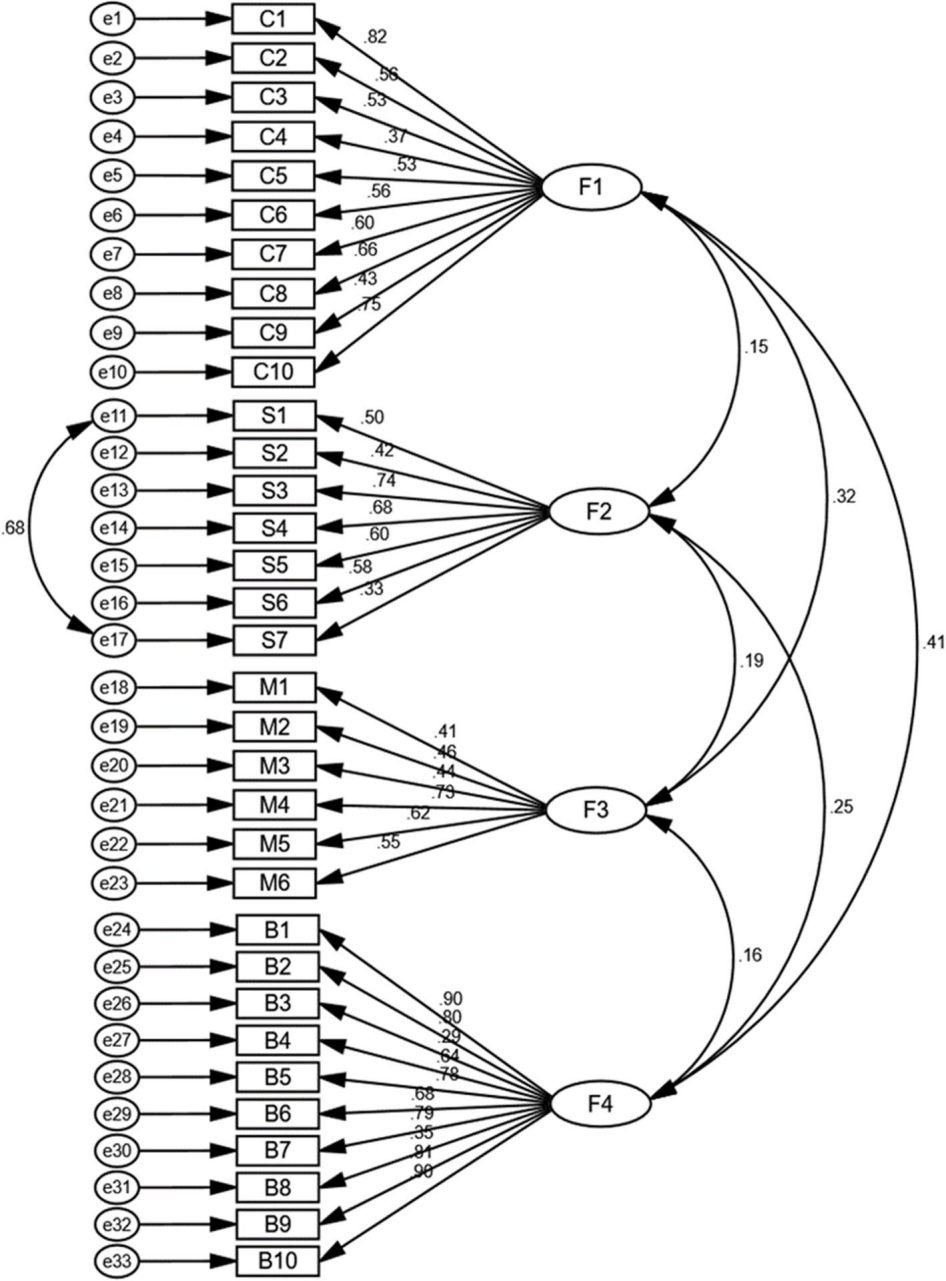
A four-factor model from confirmatory factory analysis (*n* = 215)

The results of the first CFA of this study showed that there was a high correlation between the remnants of items S1 and S7. The second CFA was performed by adding a covariance connection between the error residuals of these two items. From the fitting index values (Table 7), it can be seen that all indicators except GFI, TLI, IFI, and CFI meet the fitting requirements, indicating that the four-domain model has acceptable factor validity in the current sample (Fig. 5).

**Table 7:** Model fit results of questionnaire path analysis

Model	χ^2^/df	RMSEA	GFI	TLI	IFI	CFI	PGFI	PNFI
First CFA	2.045	0.070	0.787	0.809	0.825	0.823	0.686	0.655
Second CFA	1.781	0.060	0.806	0.857	0.870	0.868	0.701	0.689
Model Optimum Criterion	< 3	< 0.08	> 0.9	> 0.9	> 0.9	> 0.9	> 0.5	> 0.5

## Discussion

### Reliability of scale preparation

This study utilized the COM-B model to guide the development of the self-management capability, support, and motivation-behavior scale for elderly hypertension patients. As the only theory that directly relates to behavioral influencing factors and intervention strategies, the COM-B model provides clear guidance for behavior analysis and intervention design [37], making it an ideal framework for developing scale dimensions in this study.

On this basis, we drew on relevant domestic and foreign scales, guidelines, and semi-structured interviews to further compile the initial item pool of the self-management capability, support, motivation, and behavior scale of elderly hypertensive patients to ensure that the scale is scientific and practical. Thereafter, 16 experts with extensive theoretical research and practical experience in the field of hypertension were selected for two rounds of expert correspondence, and 20 community-dwelling elderly hypertensive patients were selected for a small sample pre-test. The questionnaire was revised repeatedly according to expert suggestions and small sample pre-test results to ensure the rationality and completeness of the scale. After the questionnaire survey, reliability and validity test, exploratory factor and confirmatory factor analyses of the scale items was carried out successfully, resulting in four dimensions and 33 items, which further ensured the reliability of the scale compilation.

### Scientific scale compilation

#### Expert consultation stage

This research assesses the scientific validity of Delphi expert consultation results by evaluating expert representativeness, positive degree, authority coefficient, and Kendall's W coordination coefficient. The study begins by examining expert representativeness and enthusiasm, with 16 nursing experts selected for their clinical, scientific, psychological, and educational expertise, including experts in hypertension management guideline development. All experts have worked for over 20 years, with 50% holding associate senior titles and 70% holding titles above that level, indicating strong representativeness [38]. Additionally, the first-round questionnaire achieved a 100% recovery rate, with 10 experts (67%) providing revision opinions and suggestions. The response rate of the second round of questionnaires was 94%, among which 6 experts (37.5%) put forward revision opinions and suggestions. During the second round of expert consultation, there was a high level of consistency in the expert opinions, with an enthusiasm rate of over 70% in both rounds. These results demonstrate that the experts consulted through correspondence displayed significant interest and attention to the research [39]. The authority coefficients in the two rounds were 0.87 and 0.90, respectively, demonstrating that the experts had abundant theoretical knowledge and practical experience. Furthermore, the Kendall's W values were 0.312 and 0.523 (*P* < 0.05) respectively, indicating a high level of agreement among the experts.

#### Phase of reliability analysis

In this study, we carried out an internal consistency analysis, test–retest reliability and composite reliability, to evaluate the reliability of the scale. The Cronbach’s α coefficient of the total scale was 0.867, and the Cronbach’s α coefficients of each dimension were between 0.797, 0.861, 0.701, and 0.866, respectively, which are higher than Cronbach’s α coefficients reported by Liu Ning in China [21]. The test–retest reliability coefficient of the scale at an interval of two weeks was 0.894, which indicates it has good stability over time. Composite reliability is a relatively new evaluation index that overcomes the inherent limitation of Cronbach’s α coefficient by allowing correlated and unequal errors and varying load values of potential variables for each item. In this study, the combined reliability of all 4 dimensions is higher than 0.750, which falls within the acceptable range of combined reliability. Therefore, it can be concluded that the measurement reliability of potential variables in the scale is good [40].

#### Phase of validity analysis

The validity of a scale refers to whether it meets the intended design objectives and indicators. The content validity was determined by inviting authoritative experts to review the item pool. The results illustrate that the content validity index was 0.940, this is higher than the content validity reported by the HPSMBRS scale in China [20], that is, 0.91. The results of exploratory factor analysis showed that the factor loadings of all the 33 items on the scale were all ≥ 0.4, and there were no multiple loadings. The four common factors could explain 71.3% of the total variation. The factor loading matrix after rotation was basically consistent with the original theoretical structure of the scale, indicating that the scale structure is reasonable. In the confirmatory factor analysis, it was found that there was a high correlation between the residuals of items S1and S7. These two items are all about the drug management measures blood pressure, a family from aspects, one is from the medical staff, interaction between them, has a high correlation [41], and by adding a covariance link between the residuals of these items, the GFI, CFI, IFL, and TLI were 0.806, 0.878, 0.870, and 0.857 respectively, which were all less than 0.9, but were also within the acceptable range [42], to further verify the construct validity of this study.

There are several limitations of the study: First, the data was self-reported by patients, which may affect the quality and accuracy of the data; Second, all the participants in this study were from the Hengyang City, and the extrapolation of the results might be affected to a certain extent; Third, the results of GFI, CFI, IFL, and TLI in the scale did not meet the best fit criteria of the model, namely < 0.9. Therefore, further research on this scale is needed to reconfirm the construct validity of the overall scale.

## Conclusion

The self-management capability, support, motivation-behaviour scale of elderly hypertensive patients compiled in this study has good reliability and validity. Each index meets the psychometric standard, which can provide an effective evaluation tool for scientific, overall, and objective assessment of the self-management level of elderly hypertensive patients. However, all the participants in this study were from a region in Hengyang. Therefore, it is necessary to extract a larger sample to analyze and test the scale items in a wider area in a future study to establish the norm of scale, making the scale more convincing and stable.

## Data Availability

The data used in the study are available from the corresponding author on reasonable request.
